# Initial misdiagnosis of melanoma located on the foot is associated with poorer prognosis

**DOI:** 10.1097/MD.0000000000004332

**Published:** 2016-07-22

**Authors:** Wiebke Sondermann, Lisa Zimmer, Dirk Schadendorf, Alexander Roesch, Joachim Klode, Joachim Dissemond

**Affiliations:** Department of Dermatology, Venerology and Allergology, University School of Medicine Essen-Duisburg, Essen, Germany.

**Keywords:** chronic wound, disease-free survival, foot, malignant melanoma, misdiagnosis, prognosis, overall survival rate

## Abstract

Acral melanoma has been reported to be associated with poorer outcomes than melanoma occurring on other cutaneous sites. It has been suggested that part of this disparity in outcomes may be related to delay in diagnosis. Therefore, we have analyzed the rate of misdiagnoses in patients with melanoma located on the foot and have characterized the influence on the clinical course and survival of the patients. A prospective, computerized melanoma database at the Skin Cancer Center of the University Hospital Essen, Germany was used to identify patients with histologically confirmed melanoma located on the foot between 2002 and July 2013 for subsequent analysis. A cohort of 151 patients diagnosed with primary melanoma located on the foot was identified. One hundred seven patients qualified for subsequent analysis. Forty-two patients were male (39.3%) and 65 (60.7%) were female; the mean age at first diagnosis was 61.6 years (median 66 years). The youngest patient was 19 years, the oldest 88 years old.

Of the 107 patients analyzed, 32 (30%) were initially misdiagnosed. Misdiagnoses included chronic wounds, nevi, hematoma, fungal infections, warts, and paronychia. Misdiagnosis caused a median delay in diagnosis of 9 months. The 5-year disease-free survival rate (47.8% vs 72.7%) and the 5-year overall survival rate (63.5% vs 88.4%) were statistically significant lower in the misdiagnosis cohort.

The awareness of potentially overlooked melanoma located on the foot has to increase among physicians.

To improve early detection and, thus, the prognosis of patients with melanoma located on the foot, taking a biopsy from any suspicious lesion should be taken into consideration as soon as possible.

## Abstract 2

Supplemental Digital Content is available in the text

## 1 Introduction

Melanoma has become a growing interdisciplinary problem in public health worldwide. According to the World Health Organization (WHO), the incidence of melanoma is increasing faster than any other cancer in the world. Melanoma is the third most common cancer in Australia and the fifth in the United States of America (USA). The American Cancer Society estimated that about 70,230 new melanomas were diagnosed in the USA in 2011, resulting in about 8790 deaths.^[1]^ Although melanoma accounts for less than 5% of skin cancer cases, it causes more than 75% of skin cancer deaths^[2]^ and, thus, represents a significant health issue and economic burden.^[3,4]^ It is well known that a high tumor thickness (Breslow depth), the histological ulceration state of the primary melanoma and increased mitotic rate are associated with a poorer prognosis.^[5]^ Up to 15% of all cutaneous melanomas are localized at the foot and ankle. Moreover melanoma is the most common neoplasm seen at feet.^[6]^

The WHO distinguishes 4 main histopathological subtypes of melanoma: superficial spreading malignant melanoma (SSM), nodular malignant melanoma (NMM), lentigo maligna melanoma (LMM), and acrolentiginous malignant melanoma (ALM).^[7,8]^ ALMs show special histological characteristics and are often equated with melanoma in acral localizations.^[9]^ About 1% to 7% of all cutaneous melanomas in Caucasians are ALM.^[10]^ In Asians, Africans, and the Middle Eastern population, ALM shows a significantly higher prevalence as compared to Caucasians and accounts for up to 70% of all melanomas.^[11,12]^ Several studies demonstrated a poorer prognosis of ALM in comparison to melanomas of other localizations.^[13,14]^ It has been discussed that this is mainly attributed to the prolonged diagnosis of ALM. The delay in diagnosis may be caused by the relative inaccessibility of the feet for self-assessment. In addition, many common skin diseases like fungal infections, warts, hematoma, or chronic wounds (e.g., in diabetes) appear at the feet and can lead to misdiagnoses. Therefore, the aim of our study was to analyze the rate and duration of misdiagnosis in patients with melanoma located on the foot and to characterize the clinical consequences.

## 2 Methods

### 2.1 Identification of patients

A prospective, computerized melanoma database at the Skin Cancer Center of the University Hospital Essen, Germany was used to identify patients with histologically confirmed melanoma located on the foot which were diagnosed between 2002 and July 2013.

Patients with acral melanomas localized at other body sites, including the hand, were excluded from the cohort as well as patients exhibiting acral melanoma metastases. Tumors were categorized as ALM or NM which were the most frequent histological subgroups. Rarer subtypes as well as samples lacking classification were grouped together in the group other. Misdiagnoses and previous treatments were identified by medical history.

This prospective, observational study was approved by the Institutional Review Board of the University of Duisburg-Essen (IRB protocol number 12-4961-BO). All patients included in the study provided written informed consent. Patient written consent was also granted for medical images published in the study.

### 2.2 Statistical evaluation

The statistical analysis was performed with SPSS (Statistical Package for Social Science, SPSS, Inc., Chicago, IL) version 22. The Chi-square test or Fisher exact test were used to evaluate relationships between categorical variables. Kaplan–Meier plots and the log-rank test were used to evaluate the relationship between the diagnosis (initially correct diagnosis vs misdiagnosis) and the outcome starting from the date of surgical melanoma resection to the date of first disease recurrence or death or the last follow-up visit, respectively. The association between misdiagnosis, Breslow depth, histology, sex, local recurrence, age, and ulceration as prognostic factors was analyzed for the clinical outcome by univariate analysis and stepwise multivariate Cox-regression analysis. Hazard ratios and 95% confidence intervals (CIs) were calculated from the Cox-regression model including all factors for multivariate analysis as a 2-sided test. Differences were regarded significant at *P* < 0.05.

## 3 Results

### 3.1 Patient characteristics and main results

A cohort of 151 patients with acral melanoma located on the foot was identified of whom 107 patients qualified for subsequent analysis (cf. Supplemental STROBE flow diagram). Forty-two patients were male (39.3%) and 65 (60.7%) were female. The female to male ratio was 1:1.6. The mean age at first diagnosis was 61.6 years (median 66 years). The youngest patient was 19 years, the oldest 88 years old. The tumor thickness varied between 0.1 and 20 mm (mean 2.4 mm, median 1.6 mm). A significant difference in tumor thickness was seen between patients with an initial misdiagnosis (mean 3.7 mm, median 3.1 mm) and patients with an initially correct diagnosis (mean 1.9 mm, median 1.1 mm) (*P* = 0.001). 39.3% of all melanomas (n = 42) were ulcerated (Table 1).

**Table 1:**
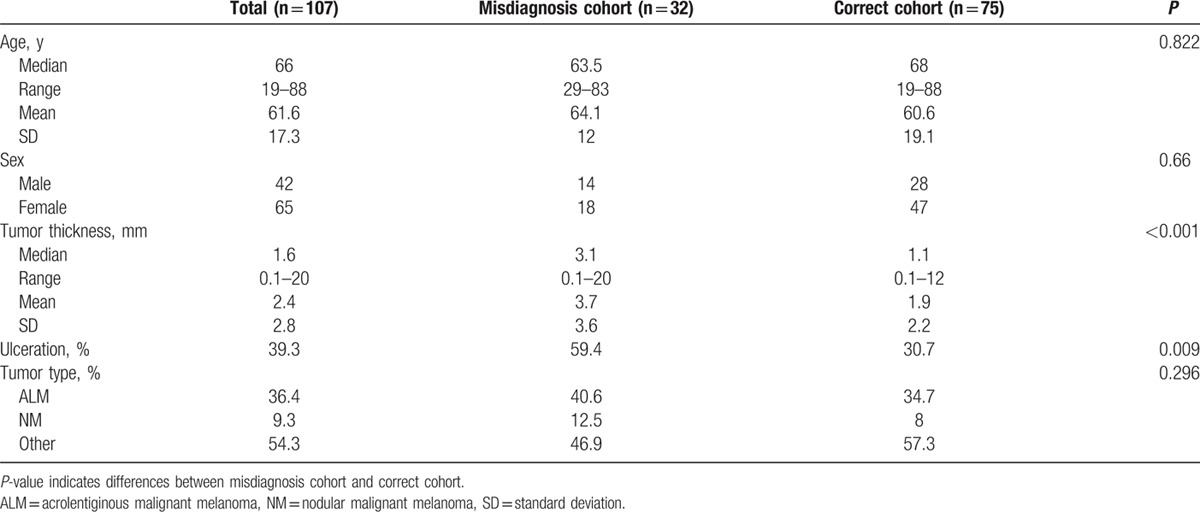
Patient characteristics.

### 3.2 Misdiagnoses and first clinical signs

In 32 of the 107 patients (30%, 18 females, 14 males), skin lesions at the feet were incorrectly diagnosed at the first medical visit. The median age for patients who were initially misdiagnosed was 63.5 years (range 29–83 years) compared with a median age of 68 years for initially correctly diagnosed patients (range 19–88 years). The most frequent misdiagnoses were wounds including diabetic foot ulcers, traumas, and peripheral arterial occlusive disease in nearly 50% of the cases (15 out of 32 patients, Table 2).

**Table 2:**
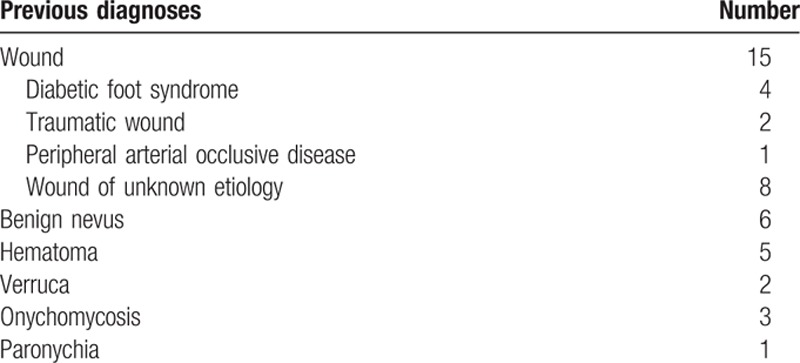
Misdiagnoses and corresponding number of patients.

At the first visit, patients typically presented with common symptoms such as painless new lesions or macules with changing color or ulcerations which were mostly interpreted as trauma or wounds (Table 3).

**Table 3:**
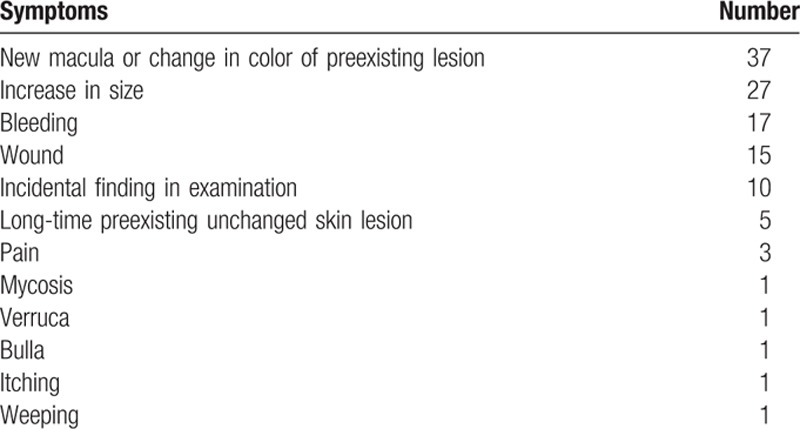
First clinical symptoms and corresponding number of patients.

### 3.3 Tumor stage at initial diagnosis of melanoma

In 104 of the 107 patients, information on the tumor stage was available at initial diagnosis of melanoma. Overall, 80 patients (76.9%) presented with local tumor disease, that is, primary melanomas up to stage IIC according to the American Joint Committee on Cancer (AJCC) 2009 classification; 24 patients (23.1%) had already developed advanced disease with lymph node and/or organ metastases (AJCC stage III–IV).^[15]^

Fifty-nine (80.8%) of the patients with an initially correct diagnosis at the first clinical visit (n = 73) were in a localized stage (AJCC stage I–II), while 14 cases (19.2%) showed advanced disease (AJCC stage III–IV). In contrast, 32.3% of the cases with an initially incorrect diagnosis (n = 31) already showed regional or distant tumor progression (AJCC stage III–IV). However, this difference was not statistically significant (*P* = 0.2).

### 3.4 Delay in diagnosis

Information on the exact time span between the first recognized symptoms and the date of melanoma diagnosis was available for 102 out of 107 patients. The median interval between the first clinical signs and the date of melanoma diagnosis was 138 days (0 to 6570 days). In the cohort of initially correctly diagnosed patients, the time span between first symptoms and melanoma diagnosis ranged between 0 and 3650 days (median 92 days). For patients with an initial misdiagnosis, the median diagnostic interval was 365 days (ranging from 91 up to 6570 days, *P* = 0.004). In patients with ulcerated melanomas, the median time span for correct diagnosis was 365 days (7–6570 days). Without ulceration, the median interval between first symptoms and melanoma diagnosis was significantly reduced to 92 days (0–3650 days, *P* = 0.02). In male patients, the time span from the first clinical symptoms until diagnosis of melanoma was 121 days (range 0–3650 days), in females 214 days (0–6570 days, *P* = 0.37).

### 3.5 Disease-free and overall survival

**Figure 1:**
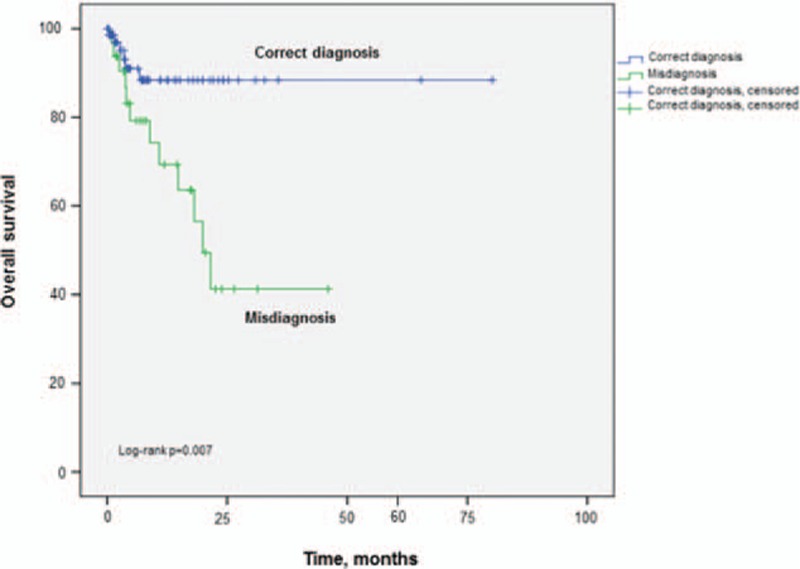
Calculated 5-year overall survival rate for the correctly diagnosed cohort versus the initially misdiagnosed cohort.

The median patient follow-up was 26.8 months (0–320.8 months). The median observational time for the misdiagnosis cohort was 34.1 months (0–184 months) and for the correctly diagnosed cohort 18.7 months (0.7–320.8 months). The disease-free survival rate and overall survival rate were analyzed for the dependency on correct or incorrect melanoma diagnosis using Kaplan–Meier survival curves (Fig. 1). Table 4 provides a summary of prognostic factors for the overall survival rate by univariate and multivariate analyses. Only the parameter “misdiagnosis” represented a statistically significant indicator for reduced survival in both the univariate (95% CI: 1.34–9.54, *P* = 0.01) and multivariate analysis (95% CI: 1.37–9.99, *P* = 0.01). The calculated 5-year disease-free survival rate was 47.8% in the misdiagnosis cohort versus 72.7% in the correctly diagnosed cohort (*P* = 0.02). The 5-year overall survival rates were 63.5% (misdiagnosis cohort) and 88.4% (correctly diagnosed cohort; *P* = 0.007, Fig. 1). The calculated overall survival rate for the whole cohort was 85.1%.

**Table 4:**
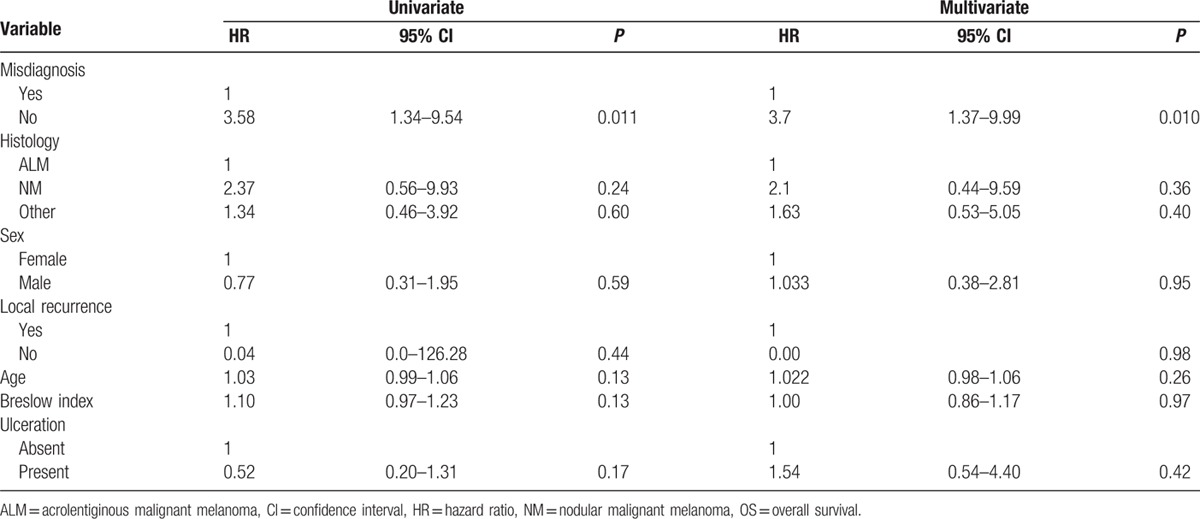
Univariate and multivariate analysis of overall survival.

## 4 Discussion

To the best of our knowledge, this is one of the largest studies on misdiagnosis of melanoma located on the foot. Our results demonstrate a median delay of melanoma diagnosis by approximately 9 months, when skin lesions on the foot have been misdiagnosed at the first medical visit. Melanomas of misdiagnosed patients showed significantly increased Breslow depth and a higher rate of ulceration than patients with an earlier diagnosis. In addition, we demonstrated that an initial misdiagnosis was associated with poorer prognosis regarding 5-year disease-free survival rate and 5-year overall survival rate.

### 4.1 Misdiagnoses and first clinical signs

In our study, 30% of the melanomas were incorrectly diagnosed at the first medical visit. This is in line with the results of other groups.^[14,16,17]^ For example, Fortin et al^[14]^ found an initial misdiagnosis rate of 25%, while Bristow and Acland^[16]^ reported a rate of incorrect diagnosis of 33%. Thus, melanomas of the feet seem to be more frequently misdiagnosed as compared to melanomas from other body sites where the overall misdiagnosis rate is approximately 10%.^[18,19]^ However, there are also reports on errors in diagnosis of melanomas not only located at the foot. For example, a recent study by Simionescu et al analyzed errors in approaches to melanoma. Among 30 patients, 36 clinical diagnostic errors were made (multiple errors were possible).^[20]^

In our cohort, the percentage of melanomas located on the foot misclassified as wounds was higher as compared to the other studies (Soon et al^[17]^: 11% wounds, Bristow and Acland^[16]^: 14% wounds). The most frequently observed clinical presentation of melanomas in our study was a new patch or a change in color of a preexisting lesion. An increase in size of a preexisting lesion was the second most common symptom reported by our patients while it was the most frequent symptom in the study by Bristow and Acland.^[16]^ In the series of Bristow and Acland^[16]^ a change of color was reported by 2 of 21 patients. Bleeding was the third most common symptom in our study and the second most reported in the study by Bristow and Acland.^[16]^

### 4.2 Tumor types

ALM was the most frequent tumor type in our study, diagnosed in 36.4% of our patients. In other studies, the frequency of ALM ranged from 29% to 67% in melanomas located at hands and feet.^[17,21–24]^

### 4.3 Tumor thickness and ulceration state

The mean tumor thickness for all patients of our study was 2.4 mm (median tumor thickness 1.6 mm), which is lower as compared to Fortin et al^[14]^ who reported a mean tumor thickness of 3.0 mm and Soon et al^[17]^ who reported a mean of 3.3 mm. The mean tumor thickness in patients with initial misdiagnosis in our study was 3.7 mm (median tumor thickness 3.1 mm) and, thus, significantly higher in comparison to patients with a correct diagnosis of melanoma (mean 1.9 mm, median 1.1 mm). Comparable data were reported by Bennett et al^[24]^ who found a mean tumor thickness for initially correct diagnosis of 2.2 mm versus 3.8 mm for patients with initial misdiagnosis. With 4.3 mm, Fortin et al^[14]^ reported an even higher mean thickness for misdiagnosed melanoma in their cohort.

Regarding all body sites, the median Breslow depth at first diagnosis in Germany is about 0.9 mm (mean 1.66 mm).^[3,25]^

In our series, we found ulcerations in 39.3% of the tumors. This is comparable to the rate of ulceration of 36% showed by Phan et al^[23]^ in a large histopathological investigation of 121 patients.

### 4.4 Tumor stage at initial diagnosis and delay in diagnosis

Overall 76.9% of the melanomas of our study were classified as local disease. Current data from the melanoma register in Tübingen, Germany indicate that 88.6% of all melanomas are diagnosed in AJCC stage I–II.^[3]^ The median delay in melanoma diagnosis of 9 months found in our study was slightly shorter as compared to results from other studies. For example, Metzger et al^[19]^ found a mean delay in diagnosis of 12 months for palmoplantar melanoma and of about 18 months for subungual melanoma. Bristow and Acland^[16]^ reported an average delay of 13.5 months.

### 4.5 Data on survival and prognostic factors

Our results suggest that the 5-year disease-free survival rate as well as the overall survival rate is significantly reduced in patients with misdiagnosis of melanoma located on the foot. The calculated 5-year overall survival rates were 63.5% in the misdiagnosis cohort versus 88.4% in the correctly diagnosed cohort.

In the study of Metzger et al^[19]^ the estimated 5-year survival rate for initially correctly diagnosed subungual melanoma was 90.9% and 68.5% for misdiagnosed cases. However, in their study, melanomas of the hand were also included. Kuchelmeister et al^[13]^ found a 5-year survival rate of 71% for ALM irrespective of the body site, while Phan et al^[26]^ reported a 5-year survival rate of 76% for a similar cohort. However, Phan and Kuchelmeister did not differentiate between initially correctly classified lesions and misdiagnosed lesions.

Only the factor “misdiagnosis” was identified in our study as statistically significant independent factor for prediction of overall survival. Unexpectedly, the histological tumor thickness was no significant discriminator. This observation is confirmed by several other studies.^[27,28]^ For example, Phan et al explained this paradox by a lower accuracy of histological tumor assessment in acral tumor locations due to the artificial fragmentation during surgical excision. Additionally, the histological tumor measurement could be less accurate in such locations because inappropriate surgical procedure are often conducted before the final excision and diagnosis.^[23]^

### 4.6 Reasons for high misdiagnosis rate in melanoma located on the foot

Cutaneous melanoma represents a tumor entity that can be early detected, by visual (self-) examination. However, considering an average age for first diagnosis of ALM of 60 to 70 years, self-examination is not always feasible due to physical limitations of the patients (reduced eyesight and mobility). Also professional examination of the tight interdigital space is hampered by limited accessibility, in particular for dermatoscopes. Furthermore, the commonly used “ABCDE” rule for self-examination is not applicable to most ALMs due to the lack of typical clinical features of pigment tumors when located at glabrous skin.^[16]^ In addition, many common skin diseases like hematoma, fungal infections, warts, or chronic wounds frequently appear at the feet and can mimic melanoma.

### 4.7 Relevance for the clinical routine

One characteristic of our study was the high rate of melanomas located on the foot which were initially misdiagnosed as wounds in the context of diabetic foot syndrome, peripheral occlusive *arterial* disease, posttraumatic ulcer, or unknown etiology. Because of the high prevalence of these diseases, especially diabetic foot syndrome and peripheral occlusive *arterial* disease, there is a high likelihood of misinterpretation of melanomas as wounds. It should be taken into account that a complete reepithelialization of wounds at the feet may take longer than 8 weeks. Nonetheless, there should be a healing tendency under adequate therapy. If this is not the case, a biopsy should be taken to histologically rule out malignancy at a maximum after 8 weeks.^[29–31]^ Lesions which are suspicious for melanoma from the beginning should be analyzed histologically as soon as possible.

### 4.8 Strengths and weaknesses of the study

The large sample size enabled us to perform an extensive analysis of the clinical misdiagnosis of melanoma located on the foot and the influence on the clinical course and survival of the patients. We have included only melanoma on the feet but not the hands, which are known to have a better prognosis because of a usually earlier recognition.

Our study results may be biased by the specialized set-up of our wound care department and, thus, patient recruitment for this study. As part of a university setting, we see selected therapy-refractory cases. This could be a confounding factor with regard to a higher rate of ulceration and more advanced tumor stages.

**Figure 2:**
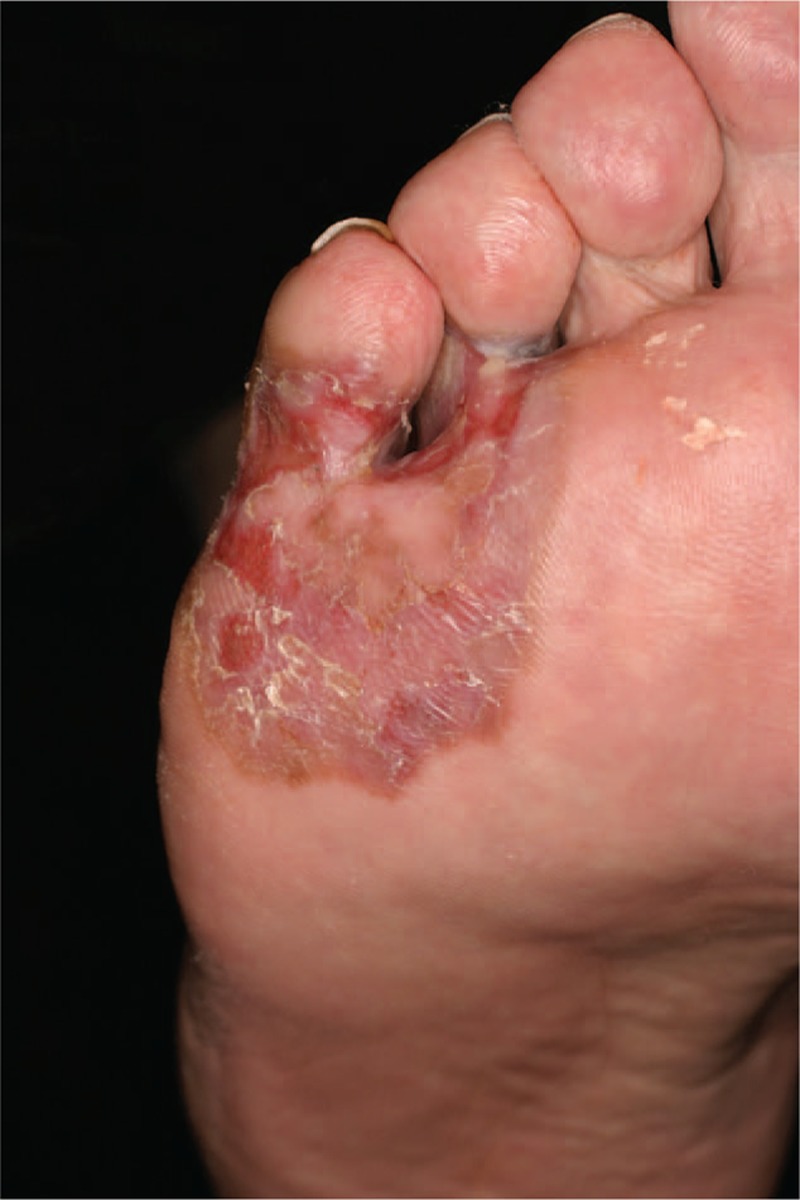
Melanoma located on the foot initially misdiagnosed as a fungal infection.

**Figure 3:**
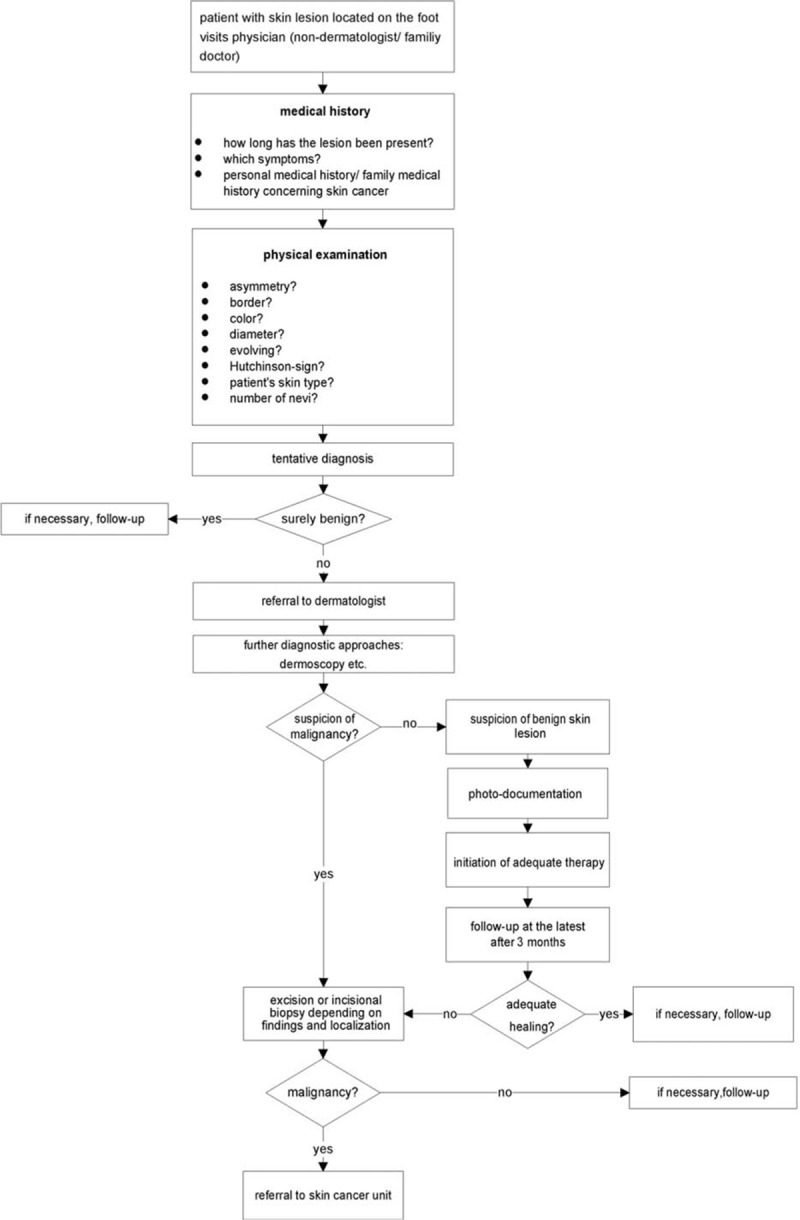
Flow chart for the diagnosis of melanoma located on the foot.

In sum, melanomas located on the foot represent diagnostic pitfalls (Fig. 2). Difficulties in (self-) examination and the occurrence of common benign differential diagnoses at this particular body site promote a considerable delay in diagnosis resulting in a poorer prognosis. In order to reduce any delay in diagnosis, we suggest a diagnostic algorithm with basic instructions also for nondermatologists for the assessment of unclear skin lesions located on the foot (Fig. 3).

## Supplementary Material

Supplemental Digital Content
