# Supplementary material for: Initial misdiagnosis of melanoma located on the foot is associated with poorer prognosis
Source: Medicine (Baltimore). 2016 Jul 22;95(29):e4332. doi: 10.1097/MD.0000000000004332 (PMC5265802; doi:10.1097/MD.0000000000004332)
Supplement: Supplemental Digital Content [file medi-95-e4332-s001.doc]

**Allocation**

**Analysis**

**Follow-Up**

**Enrollment**

Assessed for eligibility (n= 151)

Excluded (n=44)

♦  Not meeting inclusion criteria (n= 40)

♦  Declined to participate (n=2 )

♦  Other reasons (n= 2)

Analysed (n= 107)
♦ Excluded from analysis (n= 0)

Lost to follow-up (give reasons) (n= 0)

Discontinued intervention (n=0)

Allocated to intervention (n= 107)

♦ Received allocated intervention (n=107 )

♦ Did not receive allocated intervention (n= 0 )

**STROBE Statement**

Randomized (NA, only one arm)
